# Particle and Gel Characterization of Irinotecan-Loaded Double-Reverse Thermosensitive Hydrogel

**DOI:** 10.3390/polym13040551

**Published:** 2021-02-13

**Authors:** Fakhar ud Din, Sung Giu Jin, Han-Gon Choi

**Affiliations:** 1 College of Pharmacy & Institute of Pharmaceutical Science and Technology, Hanyang University, 55 Hanyangdaehak-ro, Sangnok-gu, Ansan 15588, Korea; fudin@qau.edu.pk; 2 Department of Pharmacy, Quaid-I-Azam University, Islamabad 45320, Pakistan; 3 Department of Pharmaceutical Engineering, Dankook University, 119 Dandae-ro, Dongnam-gu, Cheonan 31116, Korea

**Keywords:** irinotecan, solid lipid nanoparticles, thermosensitive nanocarrier, particle property, gel property

## Abstract

The irinotecan-loaded double-reverse thermosensitive hydrogel (DRTH) is a dispersed system of irinotecan-loaded solid lipid nanoparticles (SLN) in a thermosensitive hydrogel. To optimise the particle and gel properties of DRTHs for rectal administration of irinotecan, SLNs and DRTHs were prepared with tricaprin, triethanolamine, Tween 80, and Span 20. Among the SLNs tested, an SLN composed of 1 g irinotecan, 0.5 g lipid mixture, and 0.5 g combined surfactant gave the highest entrapment efficiency and smallest particle size. A DRTH composed of (poloxamer 407/poloxamer 188/combined surfactant/SLN dispersion/H_2_O (10/15/17/4/54%)) showed easy administration, fast gelling, and strong gel-forming in the body.

## 1. Introduction

Despite momentous advancement in the drug delivery, the novel anti-tumour modalities normally exhibit fast degradation and excretion in vivo, resulting in poor bioavailability and reduced targeting of the tumor [1]. Thus, it becomes evident that the tumor targeting additionally depends on the progression of clinically feasible drug delivery systems that are capable of proficient drug encapsulation and may ensure the transportation of pharmaceutical agents to their target cells [2]. One such strategy is the use of biomedical and biocompatible polymers, including natural and synthetic polymers (for instance, hyaluronic acid, carbopol, chitosan, poly(ethylene glycol), and poloxamers). These polymers are certainly the most suitable materials beside the lipids for developing targeted nanosystems [3,4,5]. Additionally, numerous novel nanotechnology-based polymeric drug delivery systems are presently under clinical trials for the treatment of different human tumors [6]. These nanoplatforms are already testified for their improved targeting, therapeutic efficacy, and reduced cytotoxic effects [7]. In addition to therapy, polymeric nanoplatforms demonstrated timely imaging and diagnosis of numerous medical complications [8].

Irinotecan is a water-soluble camptothecin derivative for the chemotherapy of metastatic cancer of colon and rectum [9]. It was reported that this drug has been used intravenously [10,11,12] and orally [13,14]; however, their cytotoxicity and gastrointestinal toxicity were the major clinical problems related with irinotecan, an anti-tumour drug in human subjects, resulted in its limited use [15,16,17]. Hence, to solve these problems, other alternatives should be developed.

**Figure 1:**
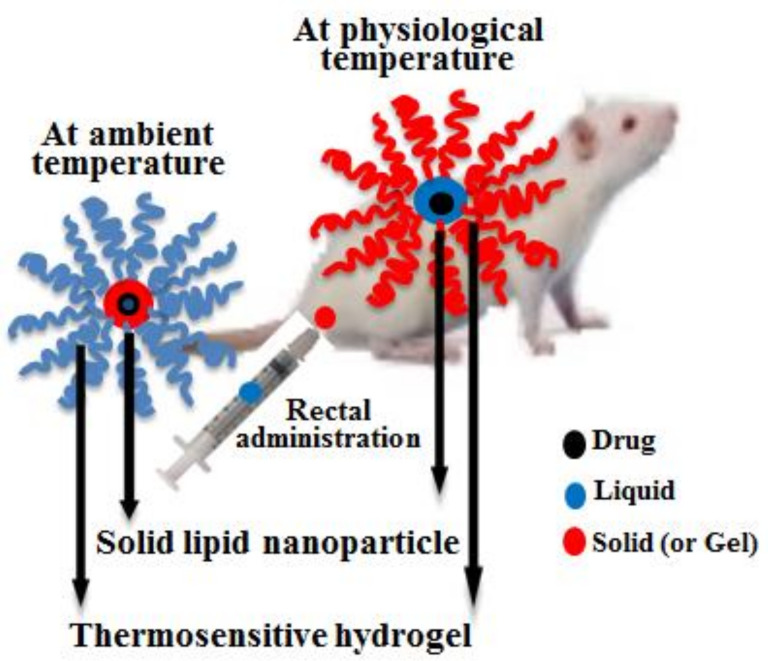
Schematic representation of drug-loaded double-reverse thermosensitive nanocarrier.

In this study, the particle and gel properties of irinotecan-loaded DRTHs system for rectal administration as an alternative have been optimised. Unlike the conventional hydrogel, this system was a dispersion that the thermosensitive hydrogel and the thermosensitive irinotecan-loaded solid lipid nanoparticles (SLN) maintained a liquid and solid form at 25 °C, respectively (Figure 1). Moreover, the former was changed to gel, and the latter was melted to liquid at 36.5 °C, respectively [18,19]. Numerous SLN dispersions and DRTHs were prepared with various amounts of ingredients, and their particle and gel properties were investigated. The conventional hydrogel system was fabricated by regulating the gelation temperature of a base, poloxamer solution which was a liquid form at 25 °C and a gel at 36.5 °C [20,21]. This hydrogel provided the enhanced bioavailability of the poorly water-soluble drugs, such as flurbiprofen, acetaminophen, levosulpiride, and insulin [20,22,23,24]. Conventional hydrogel with excellent therapeutic effect is impossible to develop most particularly when incorporated with anti-tumour drugs such as irinotecan. It is because of their severe cytotoxic effects, which are more likely to occur due to their high initial drug release (burst effect) [18,19]. Thus, irinotecan-loaded double-reverse thermosensitive hydrogels (DRTHs) with controlled drug release are proposed for solving this problem.

## 2. Materials and Methods

### 2.1. Materials

Irinotecan, Span 20, and Tween 80 (polysorbate 80) were bought from Knowshine Pharma Chemicals (Shanghai, China), Sigma Aldrich (Steinheim, Germany), and DC Chemicals (Seoul, South Korea), respectively. Poloxamer series (P-407 and P-188) were purchased from BASF (Ludwigshafen, Germany). Tricaprin and triethanolamine were obtained by Tokyo Chem. (Tokyo, Japan). All chemical reagents were used without further purification.

### 2.2. Fabrication of Irinotecan-Loaded Thermosensitive SLN

Various amounts of irinotecan, the lipid mixture (tricaprin and triethanolamine at the weight ratio of 4:1) and combined surfactant (Tween 80 and Span 20 at a weight ratio of 4:1) were gently mixed at 45 °C. These blends (1 g) were dispersed in 10 mL of deionised water, homogenised utilising a homogeniser (T-8-Ultra-Turrax, IKA; Königswinter, Germany) and a high-pressure homogeniser (Emulsiflex B15, Avestin; Ottawa, ON, Canada) using cycles of 500–1000 bar, and cooled down to 25 °C in order to produce the irinotecan-loaded SLN dispersions.

### 2.3. Particle Characterisation of Irinotecan-Loaded Thermosensitive SLN

For assessing particle size, a Zetasizer Nano ZS (Malvern Instruments; Worcestershire, UK) was used. This instrument involves software (version 6.34) and a Helium-Neon laser at the wavelength of 635 nm and static scattering angle of 90°. These SLN dispersions (10 µL) were suspended in 10 mL of deionised water and sonicated for 1 min, being carried out in triplicate [25].

For entrapment efficiency and total drug content, each SLN dispersion (1 mL) was diluted four-fold with normal saline and centrifuged at 20,000 g for 2 h at 4 °C utilising a centrifuge (Eppendorf 5430 R, Hamburg, Germany). The amount of non-loaded drug in the SLN dispersion was determined by injecting 20 µL of the subsequent solution into Capcell Pak C18 column (Shiseido, Tokyo, Japan; 4.6 mm I.D. × 250 mm, 5 μm) at 40 °C. The HPLC system (Agilent 1260 Infinity; Santa Clara, CA, USA) was utilised with G1311C 1260 and G1314B 1260 as a Quat Pump and VWD detector, respectively. The mobile phase was composed of NaH_2_PO_4_ (pH 3.1, 25 mM) and acetonitrile at the volume ratio of 50:50, respectively. With the flow rate of 1 mL/min, the eluent was checked at 254 nm. The total drug content and entrapment efficiency were determined utilising the following equations: total drug content = Ca/Ct × 100, where Ct and Ca are the theoretical and practical drug concentration, respectively; entrapment efficiency = (Wtd – Wtl)/Wtd × 100, where “Wtl” and “Wtd” are the concentration of the non-loaded and total drug in the SLN dispersion, respectively.

### 2.4. Fabrication of Arinotecan-Loaded Thermosensitive DRTHs

Numerous DRTHs were prepared with various amounts of irinotecan-loaded SLN dispersions, combined surfactant, P-407, and P-188. The combined surfactant was composed of Tween 80 and Span 20 at a weight ratio of 4:1. Their compositions are illustrated in Table 1. Momentarily, poloxamer solution was fabricated by dissolving different amounts of P-407 and P-188 in deionised water at 4 °C. The irinotecan-loaded SLN dispersions were poured to the above poloxamer solution with gentle agitation followed by being placed overnight in a refrigerator, resulting in fabricating the irinotecan-loaded DRTHs.

In addition, the morphology of the DRTHs was assessed utilising TEM (Hitachi H-7600; Tokyo, Japan) at the operation of 100 kV. In brief, DRTH was applied to a carbon-coated copper grid and allowed to stick to the carbon substrate. As a negative stain, 2% phosphotungstic acid solution was employed [26].

### 2.5. Gel Characterisation of Irinotecan-Loaded Thermosensitive DRTHs.

For assessing gelation temperature, four grams of DRTH were placed in a 10 mL transparent glass vial along with a magnetic bar (10 mm × 3 mm). This glass vial was put in the transparent glass Petri dish, and a digital thermometer (IKA ETS-D5; Guangzhou, China) was positioned in the DRTH. After constant stirring of 50–80 rpm, the system was steadily raised at a persistence rate of 1 °C/min from 20 to 40 °C. The gelation temperature, which is the temperature necessary for the DRTH to convert its state from liquid to gel, was determined as the temperature at which the rotation of the magnetic bar stopped.

Male Sprague-Dawley rats (6–8 weeks old, about 300 g) were bought from Nara Biotech. Co. (Seoul, Korea). The animals had easy access to drinking water; however, their food was stopped at least 24 h before the experimentation. They were placed under a controlled temperature and relative moisture. The procedures for the animal studies were consistent with NIH Policy and the Animal Welfare Act under the approval of the Institutional Animal Care and Use Committee (IACUC) at Hanyang University (IACUC No. 2014-0190A).

The threshold of syringeability was determined by rectal administration to the rats using a syringe with a sonde needle. It is reported earlier that a gel with viscosity 300 mPa.s at room temperature may be easily administered. In this regard, the viscosity of DRTH at 25 °C was determined and reported to be 259 mPa.s. Since it was below the threshold value, the DRTH was easy to administer without splitting the sonde needle from the syringe. Conversely, beyond the threshold, the needle became detached from the syringe, meaning that the DRTH could not be administered rectally.

After investigating the viscosity of the DRTH at 36.5 °C, the threshold of gel strength was determined as follows: each DRTH at the dose of 0.3 g/kg was rectally administered into the rat anus at the 45° slope using a sonde needle, and its leakage from the rat anus was checked for 30 min. Below the threshold, the DRTH could be released from the rat anus; however, beyond the threshold, it could not. Moreover, utilising a viscometer (Brookfield, LVDV-II+P; Middleborough, MA, USA) equipped with software (RHEOCALC; Lorch, Geremany), the rheological behaviour of the DRTH was determined at 36.5 °C. The gel strength and gelation time is the steady-state viscosity at 36.5 °C and the time needed to change the state from liquid to gel, respectively. Numerous DRTHs were prepared with various amounts of irinotecan-loaded SLN dispersions, combined surfactant, P-407, and P-188. The combined surfactant was composed of Tween 80 and Span 20 at a weight ratio of 4:1. Their compositions are illustrated in Table 1. Momentarily, poloxamer solution was fabricated by dissolving different amounts of P-407 and P-188 in deionised water at 4 °C. The irinotecan-loaded SLN dispersions were poured to the above poloxamer solution with gentle agitation. The mixture was placed overnight in a refrigerator, resulting in the fabrication of the irinotecan-loaded DRTHs.

## 3. Results and Discussion

Various polymeric drug delivery systems are currently being used to achieve the tumour targeting with enhanced therapeutic efficacy, including electrospun medicated fibers, electro-sprayed particles, biomimetic and bio-related polymeric systems, and drug-free macromolecular therapeutics [27,28,29]. All these polymeric systems have their own advantages and disadvantages. However, the electro-sprayed particles are associated with challenges such as the blockage of spinnerets in fiber production, frequent use of organic solvents, and the complexity of electrospun scaffolds to restructure the human tissues, limiting the use of electrospun products [30]. Thus, a biomimetic and bio-related polymeric system, i.e., DRTHs, was selected owing to its biodegradable and bio-absorbable polymeric nature to obtain a nontoxic platform for drug delivery with enhanced therapeutic efficacy. The DRTHs is a system composed of drug-loaded nanoparticles entrapped in thermosensitive hydrogels. The nanoparticles (SLNs) and hydrogel in the DRTHs remain respectively solid and lipid at ambient temperature, and reversely transformed to liquid and gel at physiological temperature. This behaviour of DRTHs not only controls the drug release but also results in improved bioavailability and augmented therapeutic effects. Further, this double-reverse drug delivery system helps circumvent the off-target toxicity of the chemotherapeutic agents, leading to a safe and effective anti-tumour therapy [31].

### 3.1. Irinotecan-Loaded Thermosensitive SLN

The conventional SLNs were prepared using a microemulsion technique, leading to their high entrapment of the drug and particles in the nano-sized range [32,33,34,35]. Similarly, in this study, to select the optimal composition of irinotecan-loaded thermosensitive SLNs with high entrapment and very small particle sizes, the effect of composites on the entrapment efficiency and particle size of SLNs was assessed. Priorly, various thermosensitive SLN dispersions were prepared with 0.1–1 g drug, 0.2–1 g lipid mixture, and 0.2–0.5 g combined surfactants in 10 mL deionised water. In the development of the irinotecan-loaded thermosensitive SLNs, tricaprin and triethanolamine in the weight ratio of 4:1, and Tween 80 and Span 20 at a weight ratio of 4:1 were used as a lipid mixture and combined surfactant, respectively. In the preliminary study, this lipid mixture with a melting point of almost 32 °C provided a solid/gel state at 25 °C and a liquid form in 36.5 °C [18,19]. Moreover, the amounts of composites, such as drug and the combined surfactants, hardly affected its melting point.

**Figure 2:**
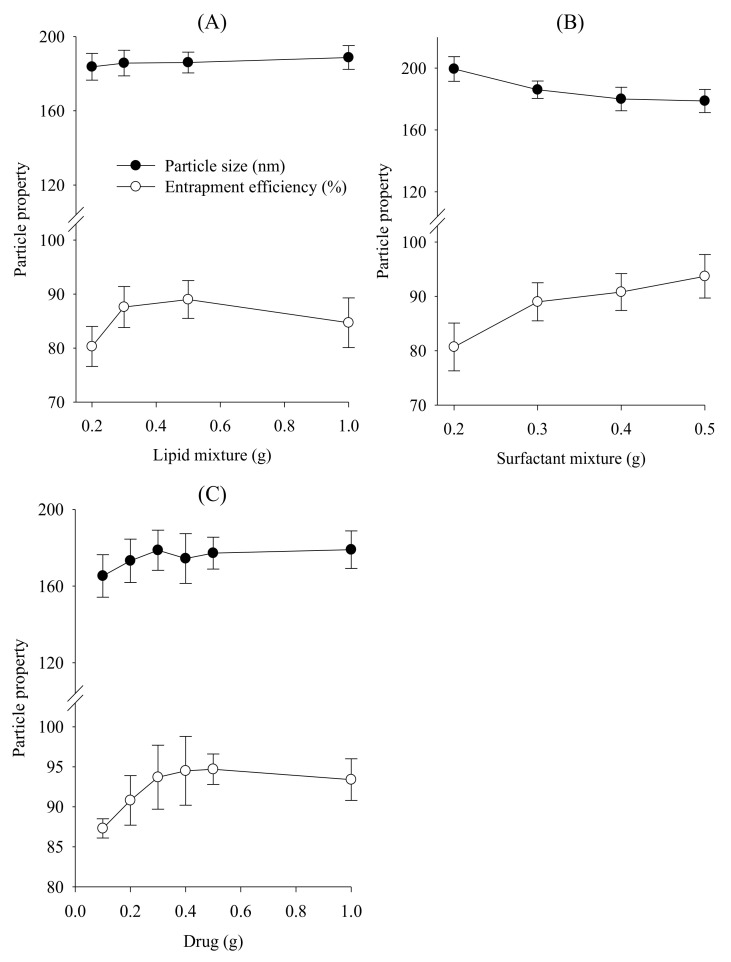
Effect of composites on the particle size and entrapment efficiency of SLN: (**A**) lipid mixture; (**B**) combined surfactants; (**C**) drug. Each value represents the mean ± S.D. (*n* = 3).

First, to investigate the effect of lipid mixture on their entrapment efficiency and particle size, numerous thermosensitive SLNs were prepared with 0.3 g drug, 0.3 g combined surfactant, and 0.2–1.0 g lipid mixture. The lipid mixture barely influenced the particle size of the formulations at the range from 184 to 189 nm (Figure 2A) [36,37]. The amount of lipid mixture from 0.2 g to 1 g increased the entrapment efficiency. In particular, the SLNs prepared with 0.5 g or 1 g lipid mixture gave significantly higher entrapment efficiency than those prepared with 0.2 g or 0.3 g lipid mixture; however, there were no significant differences in entrapment efficiency between the former groups. Thus, the amount of lipid mixture was fixed to 0.5 g.

Next, to evaluate the effect of combined surfactant, various SLNs were prepared with 0.3 g irinotecan, 0.5 g lipid mixture, and 0.2–0.5 g combined surfactant, and their properties were checked (Figure 2B). The increased concentration of surfactant leads to a slight decrease in the particle size, even if this was not significantly different. The SLNs prepared with more than 0.3 g surfactant showed significantly higher entrapment efficiency compared to that prepared with 0.2 g surfactant. Furthermore, the amount of lipid mixture from 0.3 g to 0.5 g improved the entrapment efficiency, even though there were no significance differences. The decreased particle size at relatively high concentrations of surfactant was because of the effectively reduced interfacial tension between aqueous and lipid phases, leading to the relatively small size of emulsion droplets. These emulsion droplets on cooling produced relatively small nanoparticles [33,38]. Additionally, this combined surfactant solution acted as a steric stabiliser by preventing their coalescence, leading to high drug entrapment [38,39]. Thus, the amount of combined surfactant was chosen as 0.5 g, due to the relatively small particle size and high entrapment efficiency.

Finally, the particle size and entrapment efficiency of SLNs prepared with 0.5 g lipid mixture, 0.5 g combined surfactant, and 0.1–1.0 g irinotecan were determined in order to assess the impact of the loaded drug. As shown in Figure 2C, the drug contents scarcely affected the particle size and entrapment efficiency, since the amount of combined surfactant might be enough to control these properties of SLNs [33,37]. Thus, the amount of drug was determined as 1.0 g, because the SLNs provided the maximum drug loading.

From our outcome, the SLN composed of 1 g irinotecan, 0.5 g lipid mixture, and 0.5 g combined surfactant was selected for further study, owing to its high entrapment efficiency (about 93%) and small particle size (about 180 nm).

### 3.2. Irinotecan-Loaded Thermosensitive DRTH

The irinotecan-loaded thermosensitive DRTHs were prepared with various proportions of P-407, P-188, Tween 80, and deionised water (Table 1). Their gel properties, such as gelation temperature, syringe ability, gel strength, and gelation time, were evaluated. The gelation temperature means the temperature at which the liquid phase changes to the gel state. Similar to the conventional thermosensitive hydrogels, the appropriate gelation temperature range for DRTH is 30–36 °C so that it could retain a liquid state at 25 °C and convert to gel rapidly in the rectums [20,21]. If the thermosensitive system had gelation temperatures lower than 30 °C, gelation occurred at room temperature, which led to trouble with the manufacturing, handling, and administration. Conversely, if the gelation temperatures were above 36 °C, the gels maintained a liquid state at physiological temperature, leading to leaking from the body during rectal administration. As with conventional thermosensitive hydrogels, poloxamer series, such as P-407 and P-188, were used in the fabrication of DRTH owing to their temperature-sensitivity and gelling properties [22,23,40].

Syringeability, an important factor for the rectal administration of DRTH into the body, was determined by the viscosity of DRTH at 25 °C and easy rectal administration [18]. The appropriate threshold of syringeability was confirmed by its rectal administration to rats via a syringe fitted with a sonde needle. The threshold of syringeability was described as the highest viscosity in 25 °C at which the DRTH was easily administered without separating the sonde from the syringe. The syringeability threshold for DRTH was a viscosity of about 300 mPa.s at 25 °C. Below 300 mPa.s, the DRTH was easy to administer without splitting the sonde from the syringe. Conversely, beyond 300 mPa.s, the needle became detached from the syringe, meaning that the DRTH could not be administered rectally [18].

**Figure 3:**
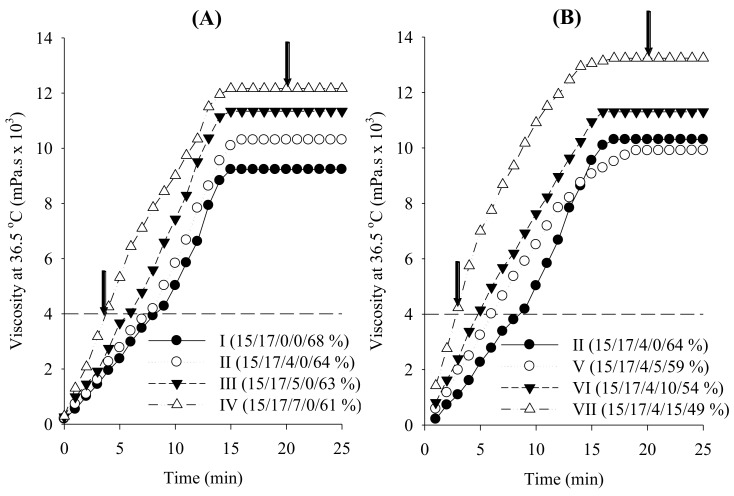
Effect of combined surfactant (**A**) and SLNs (**B**) on the rheological behaviour of double-reverse thermosensitive hydrogel (DRTHs). The DRTHs were composed of [P-407/P-188/combined surfactant/SLN dispersion/H_2_O].

To investigate the impact of combined surfactant on the gel properties of DRTHs, the DRTHs were prepared with various amounts of the combined surfactant (0–7%) and deionised water (61–68%), keeping the amounts of P-407 and P-188 at 15% and 17%, respectively (Table 1; composition I–IV). As shown in Figure 3, when the DRTHs were inserted into the body, their viscosities were gradually increased followed by constant intrinsic viscosities. Tween 80 decreased the gelation temperature and gelation time but increased the viscosity at 25 °C and gel strength (Table 1; Figure 3). The hydrogel prepared only with 15% P-407 and 17% P-188 gave the gelation temperature of 50 °C, although it showed other suitable gel properties (Figure 3A; Table 1, composition I), indicating that it was unsuitable for DRTH. Thus, Tween 80 was necessary to control the gelation temperature of DRTH and improve the stability of SLNs in the DRTH [20,41,42]. Moreover, the hydrogel with the addition of 4–7% Tween 80 gave the gelation temperature of 32–37 °C (Table 1, composition II–IV), indicating a suitability for DRTH. In this study, as SLNs gave the tendency to decrease the gelation temperature, the hydrogel prepared with 4% Tween 80 was selected due to the relatively high gelation temperature.

Subsequently, the effect of SLNs on the gel properties of DRTHs prepared with 4% Tween 80, 15% P-407, 17% P-188, 5–15% SLN dispersions, and deionised water (49–59%) was assessed (Figure 3B; Table 1, composition V–VI). All of the DRTHs prepared with SLN were easy to administer rectally due to their viscosity of less than 300 mPa.s. Moreover, they could not leak from the anus because they gave a high gel strength of above 4000 mPa.s and a fast gelation time of less than 6 min. However, the DRTHs prepared with 5–10% SLNs gave the gelation temperature in the range of 30–36 °C; however, the DRTH with 15% SLN gave a low gelation temperature below 30 °C.

The temperature-dependent gelation of poloxamer could be explained by the structural changes [22,43,44,45]. Poloxamer entities exhibit a well-arranged zigzag alignment. Upon an increase in temperature, the zigzag alignment may change to a more closed-packed meander alignment, thus resulting in an additionally viscous gel. Tween 80 and SLN with hydroxyl groups could reinforce the hydrogen bonding in the cross-linked reticular poloxamer gel as a result of its placement between the poloxamer molecules in the gel matrix, leading to an increased gel strength and shortened gelation temperature and gelation time of the DRTHs.

From our results, the DRTH (P-407/P-188/combined surfactant/SLN dispersion/H_2_O (10/15/17/4/54%)) was selected owing to its suitable gelation temperature of 32.5 ± 0.4 °C, appropriate viscosity of 259.0 ± 3.07 mPa.s at 25 °C, strong gel strength of 117.0 ± 0.6 × 102 mPa.s, and fast gelation time of 4.76 ± 0.5 min.

**Figure 4:**
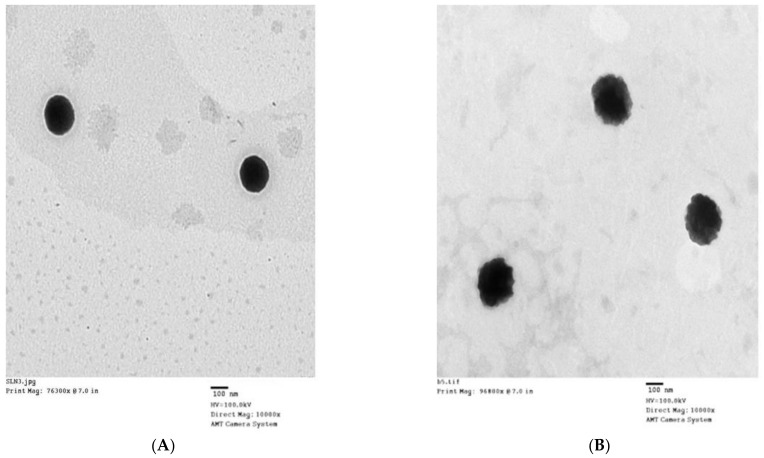
TEM of DRTH (10,000×); (**A**) 25 °C; (**B**) 37 °C.

Figure 4 shows the shape of this DRTH given by transmission electron micrograph (TEM) images. The SLNs in the DRTH illustrate a well-maintained spherical shape with a clear boundary at 25 °C (Figure 4A). However, at 36.5 °C, these SLNs gave a dubious and distorted spherical shape with a relatively large size, resulting from their melting phenomenon (Figure 4B), Moreover, a small increase in particle size at body temperature might be attributed to their practicable expulsion due to melting effect [19].

## 4. Conclusions

In the DRTH system, the ingredients, such as drug, lipid mixture, and combined surfactant, barely affected the mean particle size of SLN. The combined surfactant considerably influenced the entrapment efficiency of the drug; however, the drug and lipid mixture did not affect it significantly. Among the SLNs tested, an SLN composed of 1 g irinotecan, 0.5 g lipid mixture, and 0.5 g combined surfactant gave the highest entrapment efficiency of about 93% and the smallest particle size of about 180 nm. Moreover, Tween 80 and SLN decreased the gelation temperature and gelation time but increased the viscosity at 25 °C and gel strength of the DRTH. In particular, the DRTH composed of P-407/P-188/combined surfactant/SLN dispersion/H_2_O (10/15/17/4/54%) was easily administered in the body, quickly gelled, and formed a strong gel. Thus, further studies of the in vivo drug efficacy and toxicity evaluation will be carried out after the rectal administration of this selected DRTH in rats and tumour-bearing mice.

## Figures and Tables

**Table 1:** Gel properties of irinotecan-loaded DRTHs.

Composition	Gelatine Temperature (°C)	Viscosity at 25 °C (mPa.s)	Gel Strength (×10^2^ mPa.s)	Gelation Time (min)
I (15/17/0/0/68%)	50.0 ± 0.3	114.8 ± 4.6	92.2 ± 0.9	9.0 ± 0.4
II (15/17/4/0/64%)	37.6 ± 0.3	210.1 ± 3.3	103.0 ± 0.8	7.8 ± 0.6
III (15/17/5/0/63%)	35.1 ± 0.2	233.5 ± 3.6	111.4 ± 0.8	5.6 ± 0.6
IV (15/17/7/0/61%)	31.9 ± 1.6	287.1 ± 3.3	121.6 ± 1.0	4.0 ± 0.7
V (15/17/4/5/59%)	34.6 ± 0.2	244.8 ± 4.8	108.1 ± 0.5	5.7 ± 0.4
VI (15/17/4/10/54%)	32.5 ± 0.4	259.0 ± 3.1	117.0 ± 0.6	4.8 ± 0.5
VII (15/17/4/15/49%)	28.0 ± 0.8	284.6 ± 3.1	132.3 ± 0.5	3.0 ± 0.6

The DRTHs were composed of [P 407/P 188/combined surfactant/SLN dispersion/H_2_O]. Each value represents the mean ± S.D. (*n* = 3). SLN: solid lipid nanoparticles.

## Data Availability

The data presented in this study are available on request from the corresponding author.
